# Composite Bridge Girders Structure Health Monitoring Based on the Distributed Fiber Sensing Textile

**DOI:** 10.3390/s23104856

**Published:** 2023-05-18

**Authors:** Rui Wu, Andres Biondi, Lidan Cao, Harsh Gandhi, Sabrina Abedin, Guoqiang Cui, Tzuyang Yu, Xingwei Wang

**Affiliations:** 1 Department of Electrical and Computer Engineering, University of Massachusetts Lowell, Lowell, MA 01854, USA; rui_wu@student.uml.edu (R.W.); andres_biondivaccariello@student.uml.edu (A.B.); lidan_cao@student.uml.edu (L.C.); sabrina_abedin@student.uml.edu (S.A.); guoqiang_cui@student.uml.edu (G.C.); 2 Department of Civil & Environmental Engineering, University of Massachusetts Lowell, Lowell, MA 01854, USA; harshnareshkumar_gandhi@student.uml.edu (H.G.); tzuyang_yu@uml.edu (T.Y.)

**Keywords:** distributed fiber optic sensor, sensing textile, BOTDA, structural health monitoring

## Abstract

Distributed structure health monitoring has been a hot research topic in recent years, and optic fiber sensors are largely developed for the advantages of high sensitivity, better spatial resolution, and small sensor size. However, the limitation of fibers in installation and reliability has become one of the major drawbacks of this technology. This paper presents a fiber optic sensing textile and a new installation method inside bridge girders to address those shortcomings in fiber sensing systems. The sensing textile was utilized to monitor strain distribution in the Grist Mill Bridge located in Maine based on Brillouin Optical Time Domain Analysis (BOTDA). A modified slider was developed to increase the efficiency of installation in the confined bridge girders. The bridge girder’s strain response was successfully recorded by the sensing textile during the loading tests that involved four trucks on the bridge. The sensing textile demonstrated the capability to differentiate separated loading locations. These results demonstrate a new way of installing fiber optic sensors and the potential applications of fiber optic sensing textiles in structural health monitoring.

## 1. Introduction

Structural health monitoring (SHM) is becoming increasingly important in the management of civil engineering structures such as bridges, tunnels, and buildings. SHM is the process of continuously monitoring the structural integrity of a structure, assessing any potential damage or deterioration, and ensuring that the structure remains safe and operational [1,2,3]. It involves the use of various types of sensors to collect data on the behavior of a structure, which can then be analyzed to identify changes or anomalies that could indicate damage or potential failure. Various types of sensors are commonly used in SHM, including strain gauges, accelerometers, displacement sensors, acoustic emission sensors, and fiber optic sensors [4,5,6,7]. These sensors can be used to measure a range of parameters, including strain, vibration, displacement, temperature, and pressure. Wireless sensors based on strain gauges and accelerometers were presented to improve data acquisition structural health monitoring [8,9,10,11]. However, the traditional SHM methods typically involve periodic inspections and visual assessments, which can be time-consuming, costly, and may miss critical damage that occurs between inspections.

To overcome these limitations, advanced sensing technologies, including distributed fiber optic sensors (DFOS), have been developed for SHM [12,13,14,15,16]. Multi-wave and hybrid imaging techniques were presented to create a new direction for nondestructive monitoring [17]. However, these approaches have difficulties in providing real-time and quantitative imaging for detection. Terrestrial laser scanning (TLS) was developed for SHM, which can achieve maximum errors of about 0.1 m [18]. However, the TLS can only scan the surface of a structure and has the limitation of data processing due to the huge data size. Among these new approaches, DFOS has emerged as a promising solution that enables continuous and real-time monitoring of a structure over a large area with high spatial resolution. DFOS has been successfully applied to a range of infrastructure systems, including bridges, tunnels, railroads, and pipelines, among others [19,20,21,22]. By providing accurate and timely information on the condition of a structure, DFOS can help to improve its safety, reliability, and performance while reducing maintenance costs and downtime.

Due to the fragile nature of optical fibers, complicated installation and transportation are two of the main disadvantages of DFOS. A method of embedding optic fiber sensors into the precast piles by slotting the piles’ surface was presented [23], and this approach ensures the fiber’s safety in the structure but is only applicable to simple fiber patterns and strong structures for slotting. Embedding FOS into textiles facilitates the handling and installation of fibers, and enables seamless integration with the infrastructure [24]. The increasing utilization of smart textiles is crucial in the development of SHM systems, owing to their ability to be installed on structural shapes of any kind, including bridges with complex curvatures that pose challenges for conventional sensing systems.

Brillouin Optical Time-Domain Analysis is a state-of-the-art fiber optic sensing technique used for SHM applications [25,26,27]. BOTDA measures the temperature and strain distribution along a fiber optic cable by analyzing the Brillouin frequency shift of light scattered back from the fiber. The technique offers several advantages over other interrogation methods, making it an attractive option for SHM. One of the primary advantages of BOTDA is the capability of distributed sensing with a high spatial resolution in a large sensing range. BOTDA also provides high sensitivity, good measurement accuracy, and repeatability.

Capitalizing on these advantages, this research paper employs a distributed fiber optic sensing textile to measure variations in strain resulting from load changes, with the sensor installed inside a girder of the Grist Mill Bridge. In this sensing textile, a U-shape single-mode fiber pattern was designed to cover the sensing section twice. The BOTDA technique was utilized in this experiment for static tests. To demonstrate the sensor’s strain response, the baseline profile of strain distribution along the girder was collected. Furthermore, two loading tests with different locations on the bridge were presented. This approach reduced the labor cost of transportation and installation of fiber sensors, ensured the flexibility of researchers on sensor pattern design, and demonstrated the ability to monitor the strain distribution on bridge girders.

The paper is organized as follows. Section 2 describes the fiber optic sensing textile system, including the principle of BOTDA, fabrication, and installation of sensing textiles. In Section 3, we recorded the strain responses of the baseline test and live loading tests and compared the strain response between different loading tests. Section 4 discusses the results, reasons for Brillouin frequency’s fluctuations, and their possible solutions as a future approach. Finally, the conclusion is in Section 5.

## 2. Fiber Optic Sensing Textile System

### 2.1. BOTDA Interrogation Method

Brillouin Optical Time Domain Analysis is a fiber-optic sensing technique that measures strain or temperature changes along the length of an optical fiber. The principle of BOTDA is based on stimulated Brillouin scattering. When a laser pulse is sent through an optical fiber, it interacts with the acoustic phonons present within the fiber. These acoustic phonons are created by thermal fluctuations and mechanical deformations within the fiber [28,29,30,31,32]. The scattered light signal generated by the laser pulse contains two distinct components: the Stokes and anti-Stokes signals. The frequency difference between the Stokes and anti-Stokes signals is directly proportional to the local temperature or strain changes within the fiber. This frequency shift can be detected and analyzed to determine the location and intensity of the changes.

The frequency shift is caused by the Doppler effect, which is due to the movement of the acoustic phonons. The detected frequency shift can be used to determine the temperature or strain at that location. Since the scattered light experiences a Doppler frequency shift, the Brillouin shift $V_{B}$ can be expressed as [33]:

(1) $$V_{B}=\frac{2V_{a}n_{eff}}{\lambda_{f}}$$

where $n_{eff}$ is the effective refractive index of the sensing fiber, $\lambda_{f}$ is the optical wavelength and the $V_{a}$ is the acoustic velocity. The strain applies in the fiber and has an effect on the speed of sound, resulting in a change in Brillouin frequency. The Brillouin frequency shift (BFS) caused by strain ε at a specific temperature, T, can be given by:

(2) $$V_{B}\left(\varepsilon \right)=V_{B}\left(0\right)+\frac{d\nu_{B}\left(\varepsilon \right)}{d\varepsilon }\varepsilon$$

where $V_{B}\left(0\right)$ is the initial Brillouin frequency and is independent of strain, $V_{B}\left(\varepsilon \right)$ is the BFS introduced by the applied strain ε in the fiber, and $d\nu_{B}\left(\varepsilon \right)/d\varepsilon$ is the strain coefficient. According to previous research [34], the standard single-mode fiber’s strain and temperature coefficient were about 1 MHz/°C and 50 KHz/με at the wavelength of 1.55 μm, respectively. In this paper, BOTDA methods interrogated the sensing textile and the spatial resolution achieved by the system is 1 m.

Due to the temperature variation, the deformation of fiber material can also apply strain and result in Brillouin frequency shifts. The Brillouin shift can be expressed as:

(3) $$dV_{b}=\frac{dV_{b}}{dt}dt+\frac{dV_{b}}{d\varepsilon }d\varepsilon$$

where $\frac{dV_{b}}{dt}\;$ and $\frac{dV_{b}}{d\varepsilon }$ are the temperature and strain Brillouin coefficient, respectively. According to the previous research, the bare fiber’s Brillouin temperature coefficient is 1 MHz/°C. However, in the sensing textile, the jacket single-mode fiber was used instead of bare fibers. The thermal effect on the cladding and jacketing part needs to be considered. To demonstrate temperature compensation, the temperature coefficient calibration test was conducted in our lab. We placed a 180 m jacket single-mode fiber (SMF-1300/1550-9/125-1TBYL-L) in a temperature-controlled water tank and collected the Brillouin frequency for 22 h. The result of the calibration test was 3.5 MHz/°C and this was used in temperature compensation of thermal strain applied on the sensing textile. The total strain was measured in the section of the cable line and the final compensated strain measurement is obtained by subtracting thermal strain from the total strain. All the experimental data discussed in the following sections were temperature compensated.

### 2.2. Sensing Textile Design and Fabrication

**Figure 1:**
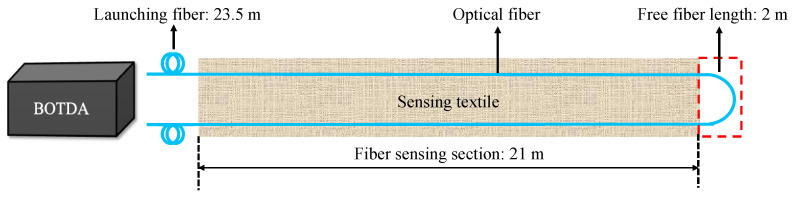
Schematic of the fiber optic sensing textile design.

The sensing textile was designed to collect strain data, using a U-shaped sensing fiber design to get a reference sensing section. This design was depicted in Figure 1, where the blue line represents the single-mode fiber for BOTDA. Both sensing sections were 21 m long, and the 23.5 m launching fibers were spliced at the starting and ending point of the textile to connect to our demodulation machine above the bridge. At the other end of the textile, there was a non-strain section of 2 m, which was not fixed by epoxy and placed outside the textile. This configuration allowed for efficient installation inside the bridge girders, providing protection to the fiber sensors. Typically, bare fiber sensors are challenging to fix on flat surfaces and require meticulous alignment of tiny fibers. However, by embedding the fiber into the textile, the installation process was simplified, and a more precise alignment was achieved. This design also allowed for efficient installation, ensuring accurate data collection and reliable protection for the fiber sensors.

To embed the fiber, an affordable reinforcing fabric by Saint-Gobain (Courbevoie, France) (XP414 laid scrim) was utilized. This particular textile was created through a chemical bonding process that interlocked a continuous filament yarn within an open mesh structure [19]. The fiber was then stitched into the fabric using an embroidery ZSK machine. This machine offered a program that allowed for different fiber patterns, enabling precise size control for all parameters. By using this approach, the fiber was seamlessly integrated into the fabric, ensuring a robust and reliable sensing textile. The sensing textile also provides an easy method of storage and transportation for fiber optic sensors. Three different fiber embedding processes have been studied, and how the different woven patterns affected the fiber sensors has been presented in [35].

### 2.3. Sensing Textile Instllation

**Figure 2:**
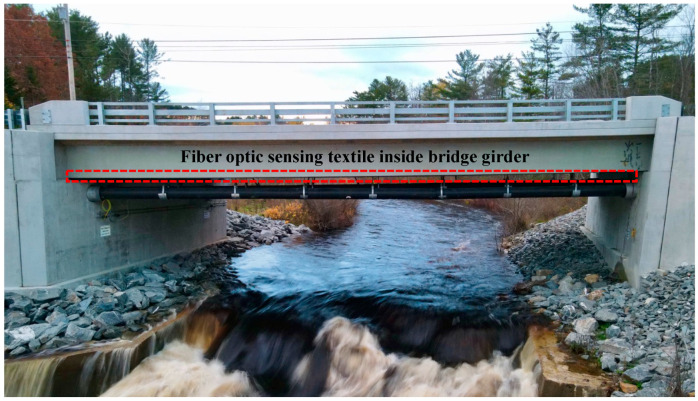
A picture of the Grist Mill Bridge.

The sensing textile was deployed at Grist Mill Bridge, situated in Hampden, Maine, as illustrated in Figure 2. This bridge is constructed using five fiber-reinforced polymer tube girders and a composite concrete deck, spanning 22.9 m. The sensing textile was installed inside the bridge girders before the girders were placed under the deck.

**Figure 3:**
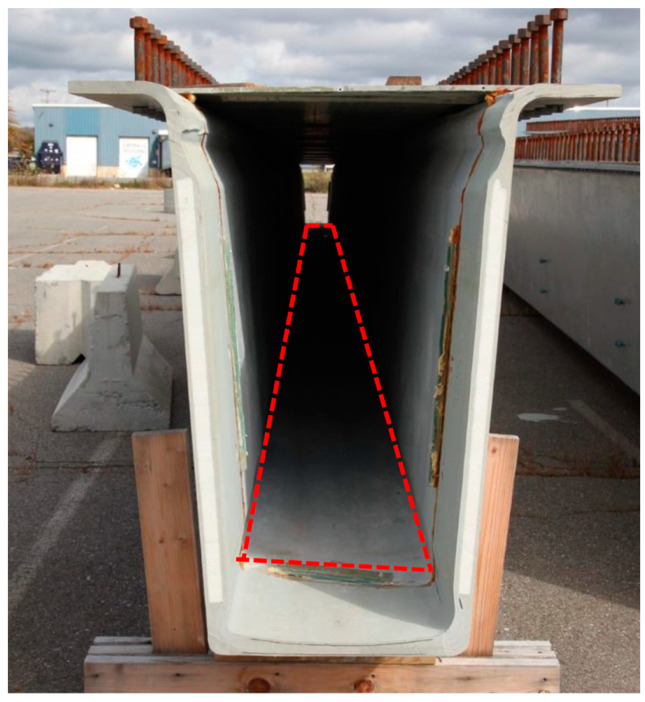
A picture of one bridge girder. The red mark indicates the location of the sensing textile.

Figure 3 shows a picture of the bridge girder. Before constructing the bridge, the engineers installed the fiber sensor, and they formed the girder using fiber-reinforced polymer. As you can see in the picture, the girder is a long and has a narrow tube shape, and the size of each girder cavity is 27 × 59 cm [36], and the installation of the sensing textile was a challenging task. The confined space limited the movement inside the girder and reduced the quality of the installation. The length of the bridge pier has reached 22 m, which further increases the difficulty of installing the textile.

**Figure 4:**
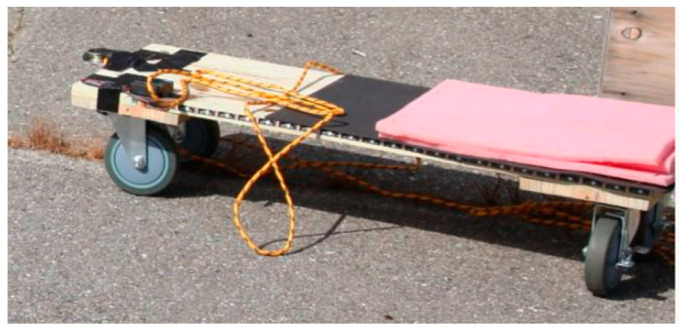
The Lab refit installation slider.

To address these problems and enhance the efficiency of the installation inside the small bridge girder, we designed and fabricated an installation slider in the lab, as shown in Figure 4. A piece of wood plate and four wheels formed the body of the slider. The right part of the slider was covered by a sponge layer to provide more support when we sit on the slider. The other half of the slider was used to place all kinds of tools we needed, such as roller brushes, a tray for brushes, gloves, and so on. We designed a small cart on the rope connected to our slider, and we used the small slider for transporting the supplies and refilling the epoxy in the tray. The sensing textile was rolled up on one roll and was easy to transport and install.

**Figure 5:**
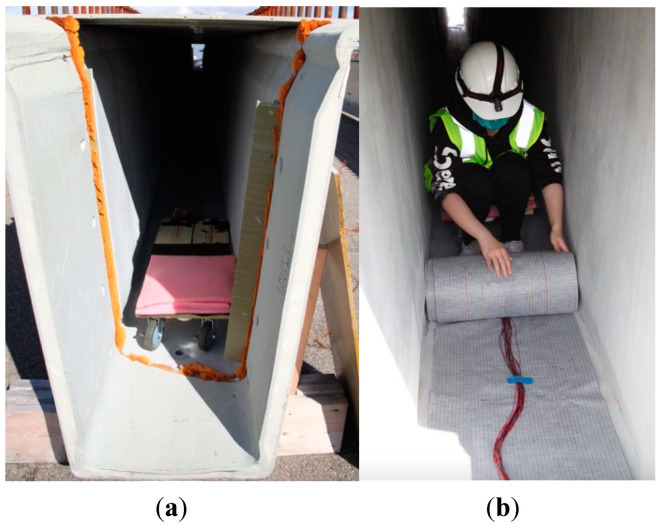
Pictures of fiber optic sensing textile installation. (**a**) The modified slider fits inside the girder. (**b**) One of our group members was unrolling the sensing textile inside the girder.

The process began by placing a slider in the girder and securing it in place with epoxy. One member of the research team then entered the girder and unrolled the sensing textile onto the epoxy-coated surface of the girder. The epoxy was carefully distributed using a brush to ensure even coverage, and any bubbles presented were removed using plastic scraper blades. This process was repeated until the entire bottom surface of the girder was covered with the sensing textile. To facilitate the epoxy refilling process, a small cart was utilized to transport supplies to the team member working within the girder. As seen in Figure 5a, the modified slider was designed to fit within the girder and allow for smooth movement without misalignment. The picture in Figure 5b shows the unrolling process when one group member was working inside the girder.

## 3. Results

### 3.1. Baseline Test on Grist Mill Bridge

**Figure 6:**
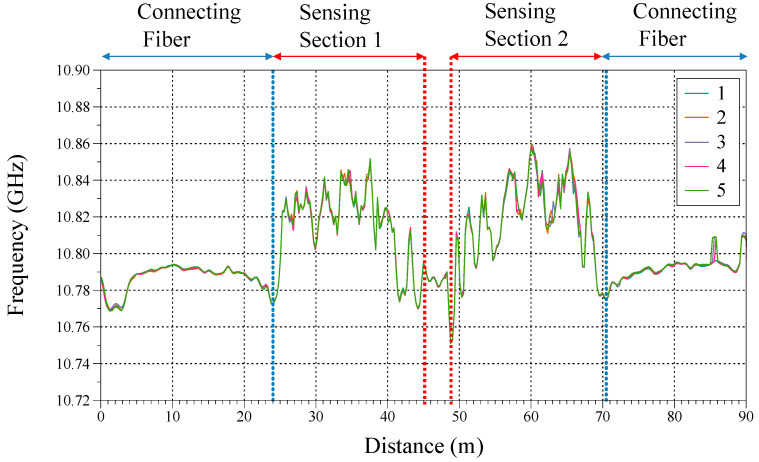
Baseline frequency response on Grist Mill Bridge without loading.

To collect the strain distribution on the Grist Mill Bridge, a field test was conducted on 31 December 2020. The output Brillouin frequency of the bridge without any loading was collected as the baseline. Five continuous sets of data were recorded to verify the stability of the fiber optic sensing system, as shown in Figure 6. All five sets of data show a good agreement, which indicates the good stability of this fiber-sensing textile. In Figure 6, the first and last parts were the launching fibers connecting the sensing textile with the BOTDA machine (DITEST Interrogator UM-031, Omnisens, Morges, Switzerland). The sensing fibers were marked as ‘Sensing Section 1′ and ‘Sensing Section 2′, and these parts were both 21 m. Between the sensing sections was a 4 m long free fiber.

### 3.2. Loading Test on Grist Mill Bridge

**Figure 7:**
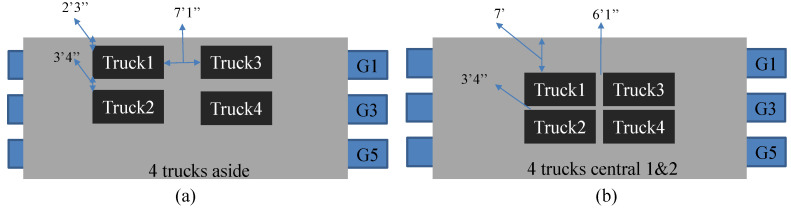
Schemes of the loading tests. (**a**) The first test of four trucks located close to one side of the bridge. (**b**) Second and third tests of four trucks located in the center of the bridge.

After collecting the baseline Brillouin frequency response of the bridge, we demonstrated two different live-loading tests by locating four overloaded dump trucks in different positions on the bridge. The schematic of the loading tests was shown in Figure 7. Black cubes in the schematic indicate the trucks, and in the first test, four trucks stopped near bridge girder 1 (G1) where our sensing textile was installed. In the second test, all trucks stopped in the center of the bridge and created different strain responses on bridge girders. The total weight of the trucks was 1100 kN and the distributed longitudinal strain data on G1 were collected by the fiber optic sensing textile. According to the schedule from Maine DOT, we were able to collect one set of data in the first test, and two sets of data in the second test.

**Figure 8:**
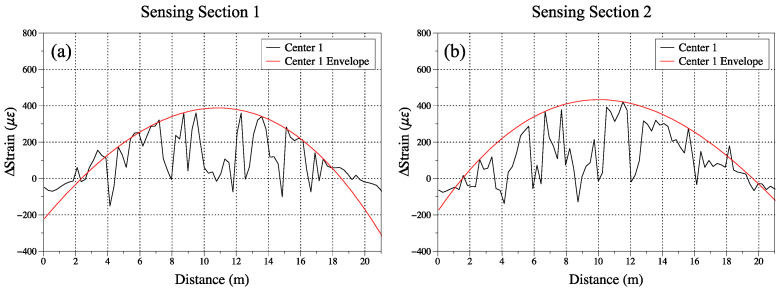

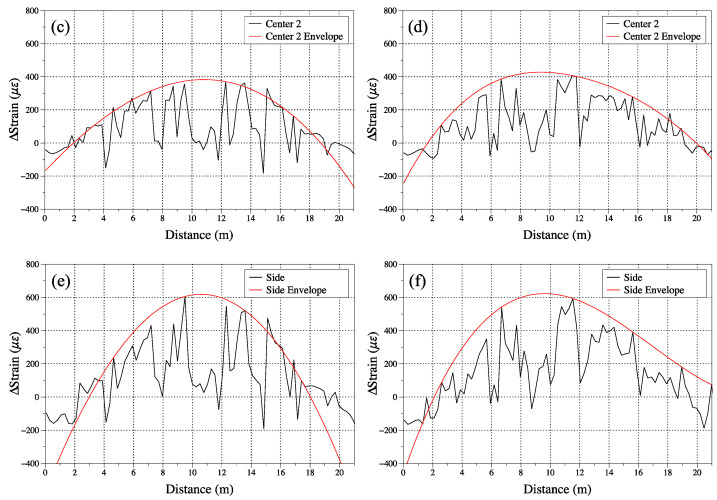
Strain distribution of different loading tests. (**a**,**b**) Experiment results of sensing Sections 1 and 2 on the first loading test with the trucks located in the center of the bridge. (**c**,**d**) Experiment results of the sensing Sections 1 and 2 on the second loading test with the trucks located in the center of the bridge. (**e**,**f**) Experiment results of sensing Section 1 and 2 on the loading test with the trucks located on one side of the bridge.

Figure 8 is an overall picture of the data collected from the loading tests. The left roll of Figure 8 shows the strain distribution from sensing Section 1 and the right roll shows the results from Section 2. Theoretically, Sections 1 and 2 should have symmetric experimental results. However, in practice, during the installation process of the sensing textile, we found that due to the inability to achieve uniform epoxy coating, there were different stress distribution profiles between Sections 1 and 2. So we can analyze the data separately from Sections 1 and 2 in different loading tests. The spikes and fluctuation in all data were due to the limitation of 1 m spatial resolution of the BOTDA and the uneven epoxy between, and the textile from the installation process also introduced some noise into the system. To minimize the impact of those limitations and noises, the envelope of the plots based on a moving average of five adjacent data points was extracted. The envelopes can better indicate the pattern of strain differences between the loading test and the baseline. The live loading on the center of the bridge can be simplified as a three-point bending test. The envelopes of the output indicated the strain data follows a Gaussian distribution that agrees with the loading response of the bridge. The unexpected valleys in the plots may be caused by the air bubbles under the sensing fiber. The installation performance was difficult to inspect after the bridge was assembled.

**Figure 9:**
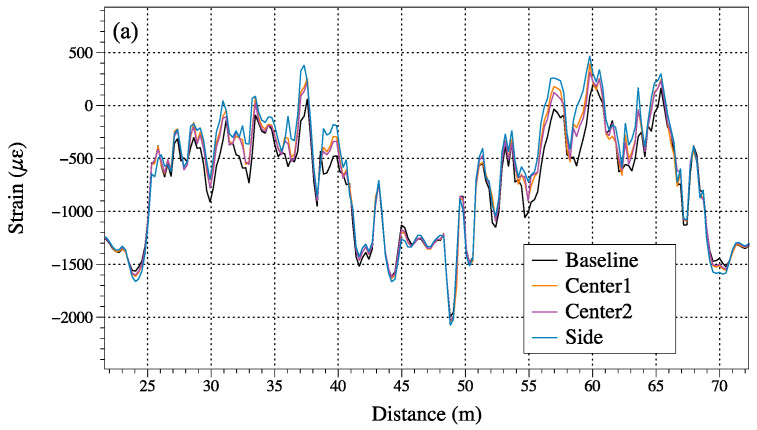

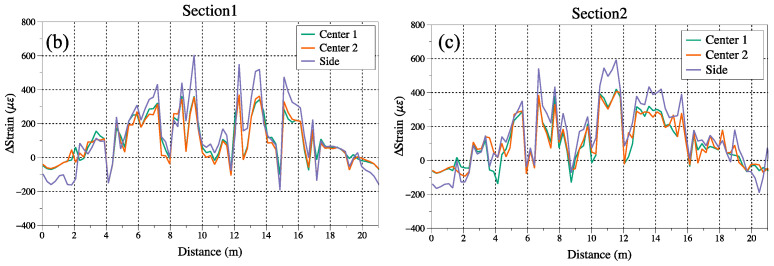
Comparison of different loading test results on sensing Section 1 and 2. (**a**) Strain distribution results of Baseline and loading tests. (**b**) Strain difference between loading tests and baseline in Section 1. (**c**) Strain difference between loading tests and baseline in Section 2.

Figure 9a shows the collected results of Baseline and three loading tests. The loading test can be differentiated from the strain distribution. We can observe from the plots that the strain differences were larger in the center and smaller in the edges. This indicated that when the bridge was applied with loading, the center part had a larger displacement than the edges. The difference between the two loading tests with baseline was shown in Figure 9b,c. In the first test, the loading trucks were located closer to the position of G1 where the sensing textile was located. It shows a higher maximum strain response of 600 με, and the maximum strain response of trucks in the center of the bridge was around 400 με. The two times of the tests under the same position of trucks in the center have a good agreement with each other in strain distribution. The difference between these two tests under the same location may be caused by the system noise.

## 4. Discussion

From Figure 8 and Figure 9, the plots show some small spikes in the sensing section may be caused by several elements. The first is the unevenly applied epoxy, which will cause pre-train on the fiber sensors. The second is the limitation of the spatial resolution of the BOTDA demodulation system. The spatial resolution is 1 m in our presented sensing textile, and some noise would be introduced below that range. The system’s scanning step frequency is the other parameter that influences the system’s accuracy and repeatability. Smaller step frequencies can output better data accuracy; however, this will increase the scanning time of the system. For example, in our baseline test, we choose a step frequency of 0.5 MHz and the scanning time was around the 20 s. If we wanted to achieve a smaller step frequency of 0.1 MHz and the scanning time was over 100 s, this would not be suitable for the live loading test according to the time schedule of the trucks.

Figure 9 shows the results of our analysis, and we observed some unexpected valleys in certain positions, such as at 4 m, 12 m, and 15 m of Section 1. We hypothesized that these positions may have been covered by air bubbles or an unfixed epoxy, which can cause the sensing fibers to deform and cause inaccurate readings. The presence of such defects may be attributed to errors in the installation process or could be due to the intrinsic properties of the materials used. Notably, the strain changes observed in these sections were minimal between the baseline frequency data and loading tests. This suggests that these positions may have been free from strain, despite the presence of the defects. It is possible that the installation process was limited in its ability to induce strain in these positions, which could have resulted in the observed strain change being negligible.

To improve the process of applying epoxy, we could use more tools in the future. For example, wider brushes to spread epoxy and scraper blades to remove bubbles. The sensing surface should also be suitably treated to ensure that it is clean and flat. The textile can choose some thicker material to improve the stiffness. As the limitation of spatial resolution of the BOTDA system, we could use a better technique, such as Optical Frequency Domain Reflectometry (OFDR), to increase the spatial resolution and the sampling rate. However, the sensing range of the OFDR system in our lab was limited to 50 m while the presented U-shape sensing textile system required a length of 91 m in total.

During field tests of the installation of fiber sensing textiles, we encountered a challenge with storing the fiber connectors. While the sensor was placed inside the girder, the connectors needed to be kept outside for easy connection to our demodulation system during testing. However, this exposed them to a harsh environment, leading to aging and damage. To address this, we developed a plug-and-play fiber connecting box that integrates different types of connectors for the sensing fiber and DAQ for strain gages. The extra launching fiber can be looped and stored inside the box, which provides more protection for the fibers in different environmental conditions. The connecting box will be installed later after the permission of Maine DOT. This solution greatly improves the durability of the fiber connecting parts and provides better protection for the fibers in the long term. By utilizing the connecting box, we not only enhance the protection of the fibers but also greatly improve the convenience of use.

The most challenging problem in scaling this technology for larger structures would be the quality of the installation. The system’s performance largely depends on the quality of the installation, and this would prove time-consuming and increase labor costs in larger structures. Advanced planning and reserving fiber installation locations can greatly reduce potential installation issues and improve installation quality, thereby better enabling the application of fiber sensing textiles in structural health monitoring.

## 5. Conclusions

This research paper proposes an innovative approach to enhancing the performance of distributed structural health monitoring systems. The study introduces a novel installation process and a newly designed fiber optic sensor embedded in textile materials. This method aims to improve the durability of the sensing system and enhance its transportability. In particular, the multi-purpose slider has been modified to address the challenges associated with installing the sensing textile in narrow bridge girders. By reducing the working hours of the installation process, this approach offers a more efficient and effective way to monitor structural health.

To evaluate the effectiveness of the proposed system, the strain responses of a bridge girder under various live loadings were investigated. The U-shaped sensing textile was tested at Grist Mill Bridge in Maine, and the results demonstrated its ability to collect the strain distribution of the bridge girder. Furthermore, the sensing textile differentiated the live loading provided by four trucks in separate locations. The sensing textile system can provide a 1 m spatial resolution and is suitable for large-scale structure monitoring.

This research presents a comprehensive approach to the development of fiber optic sensing systems for structural health monitoring. The novel installation process and the specially designed sensing textile provide an effective solution for monitoring the performance of bridge structures under heavy loads. This study’s findings demonstrate the potential of this approach to advance state-of-the-art approaches in distributed structural health monitoring and promote the safety and reliability of our critical infrastructure.

## Data Availability

Not applicable.
